# The first European woolly rhinoceros mitogenomes, retrieved from cave hyena coprolites, suggest long-term phylogeographic differentiation

**DOI:** 10.1098/rsbl.2023.0343

**Published:** 2023-11-01

**Authors:** P. A. Seeber, Z. Palmer, A. Schmidt, A. Chagas, K. Kitagawa, E. Marinova-Wolff, Y. Tafelmaier, L. S. Epp

**Affiliations:** ^1^ Limnological Institute, University of Konstanz, Konstanz, Germany; ^2^ Senckenberg Centre for Human Evolution and Palaeoenvironment, University of Tübingen, Tubingen, Germany; ^3^ Institute of Archaeological Sciences, Department of Geosciences, University of Tübingen, Tubingen, Germany; ^4^ Laboratory for Archaeobotany Baden-Württemberg, State Office for Cultural Heritage, Gaienhofen-Hemmenhofen, Germany; ^5^ State Office for Cultural Heritage Baden-Württemberg, Palaeolithic & Mesolithic Research Unit, Gaienhofen-Hemmenhofen, Germany

**Keywords:** ancient DNA, predator, prey, Hyaenidae, Rhinocerotidae, mammal

## Abstract

The woolly rhinoceros (*Coelodonta antiquitatis*) is an iconic species of the Eurasian Pleistocene megafauna, which was abundant in Eurasia in the Pleistocene until its demise beginning approximately 10 000 years ago. Despite the early recovery of several specimens from well-known European archaeological sites, including its type specimen (Blumenbach 1799), no genomes of European populations were available so far, and all available genomic data originated exclusively from Siberian populations. Using coprolites of cave hyenas (*Crocuta crocuta spelea*) recovered from Middle Palaeolithic layers of two caves in Germany (Bockstein-Loch and Hohlenstein-Stadel), we isolated and enriched predator and prey DNA to assemble the first European woolly rhinoceros mitogenomes, in addition to cave hyena mitogenomes. Both coprolite samples produced copious sequences assigned to *C. crocuta* (27% and 59% mitogenome coverage, respectively) and woolly rhinoceros (*Coelodonta antiquitatis*; 27% and 81% coverage, respectively). The sequences suggested considerable DNA degradation, which may limit the conclusions to be drawn; however, the mitogenomes of European woolly rhinoceros are genetically distinct from the Siberian woolly rhinoceros, and analyses of the more complete mitogenome suggest a split of the populations potentially coinciding with the earliest fossil records of woolly rhinoceros in Europe.

## 1. Introduction

Reconstructing past ecosystems and identifying changes in genomes of extinct populations through ancient DNA (aDNA) may help retrace population dynamics, and phylogeography and evolutionary changes. The genomic Pleistocene macro- and megafauna record of central Europe is limited to date, in part due to less suitable conditions for long-term DNA preservation in the environment, as opposed to e.g. permafrost. Predator coprolites preserved in caves are a valuable source for gathering genomic information on the individual predators and their prey.

The extinct cave hyena is a conspecific to the extant spotted hyena (occurring exclusively in sub-Saharan Africa), which ranged from the Iberian Peninsula to Asia during the Pleistocene until its extirpation in Europe approximately 14k years ago [1,2]. Previous studies confirmed a lack of divergence from extant conspecifics, suggesting considerable migration during the Pleistocene and the Holocene [3,4], and several mitogenomes of cave hyenas from European populations have been assembled for phylogenetic studies [5,6]. The prey of cave hyenas included large herbivores such as the woolly rhinoceros (*Coelodonta antiquitatis*), as evidenced by numerous macrofossil findings in European caves [7]. The woolly rhinoceros was a cold-adapted megaherbivore, which was abundant from western Europe to north-east Siberia during the Middle to Late Pleistocene [8]. Its fossil history suggests that, originating north of the Himalayan–Tibetan uplift around 2.5 Myr BP, *Coelodonta* spread westwards to enter Europe in the particularly cold and arid conditions of MIS12. The earliest immigration of *Coelodonta* into Eastern and Central Europe is documented by a number of finds dated to approximately 460–400 kyr BP [8,9] from specimens that are morphologically distinct from Late Pleistocene *C. antiquitatis.* Initially assigned to a separate species, C. *tologoijensis* [8], recent phylogenetic analyses of morphological characters imply inclusion in a subspecies, *C. antiquitatis praecursor* [10]. Irrespective of this placement, the temporal and spatial distribution of the fossils, and their morphological changes across the Middle and Late Pleistocene suggest repeated range expansions and immigration of *Coelodonta* into Central and Western Europe during successive cold periods. However, despite the wide distribution of this species throughout northern Eurasia and numerous findings of remains in western Europe, comparably little genomic information is available to investigate this. All currently published mitogenomic data of woolly rhinoceroses stem from Siberian findings [11], whereas no mitogenome assemblies of European woolly rhinos are available to date, and most molecular genetic studies on woolly rhinoceroses from Europe were restricted to few short markers [12].

## 2. Methods

Using a dendrocorer, we produced sample material from inside the two cave hyena coprolites retrieved from Middle Palaeolithic layers of two cave sites in the Swabian Jura (Hohlenstein-Stadel and Bockstein-Loch; samples referred to as HST3168 and BSVK22), Germany. We extracted DNA from the sample material (113 mg of HST3168 and 104 mg of BSVK22) as described previously ([13]; method ‘D’), with some modifications (detailed in the supplementary information). Genomic libraries were produced as described previously [14], with some modifications, using New England Biolabs reagent kits for Illumina libraries (New England Biolabs, Ipswitch, MA, USA and Qiagen, Hilden, Germany). Mammalian mitogenomic DNA was enriched using a custom-designed RNA bait panel (MYbaits, Daicel Arbor Biosciences, Ann Arbor, MI, USA) targeting a range of terrestrial mammals [15] (electronic supplementary materials). Enriched libraries were pooled at equal concentrations and were sequenced on an Illumina NovaSeq platform (Illumina, San Diego, CA, USA) with an SP Flow Cell (2 × 150 bp paired end), and from the generated sequences, we assembled mitogenomes of woolly rhinoceroses and cave hyenas.

## 3. Results and discussion

**Figure 1.**
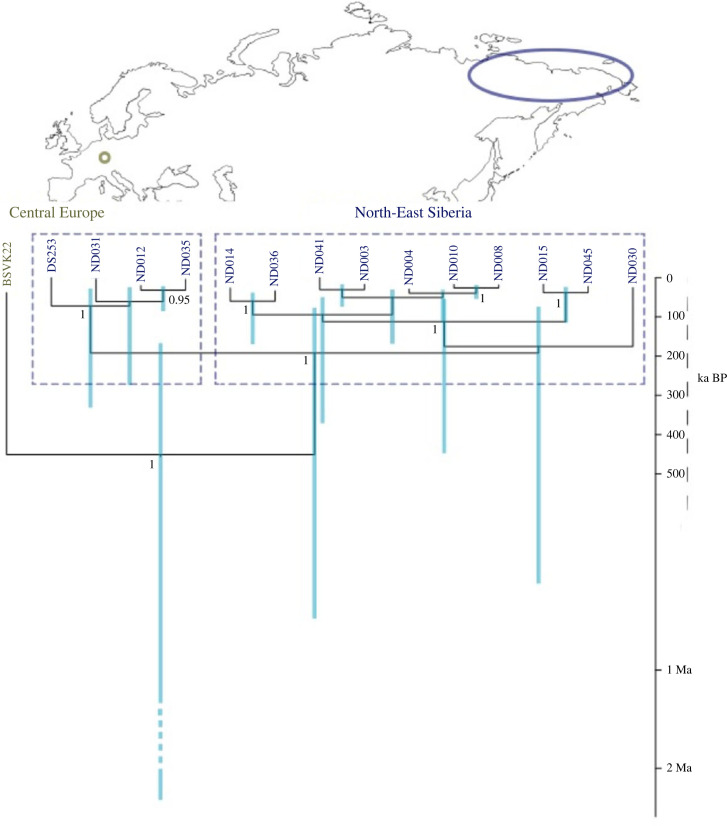
Bayesian phylogeny of the sequence from Bockstein-Loch (BSVK22) in relation to previously published sequences of woolly rhinoceroses (*Coeleodonta antiquitatis*) from Eastern Siberia [11]. Support values greater than 0.95 are shown.

The enriched coprolite libraries produced 91 873 020 and 61 090 867 raw reads, respectively. Reads assigned to taxa at a higher rank than genus, as well as reads assigned to a taxon with fewer than 1000 reads in total were disregarded, and the remaining reads (31 577 and 445 355, respectively) were exclusively assigned to *Crocuta crocuta* and *Coelodonta antiquitatis*. The extraction and library blanks produced 10 360 and 16 968 raw reads, respectively, and no reads from these libraries remained after filtering, mapping, and taxonomic assignment. The length distribution of the reads indicated considerable fragmentation, with 39 bp average length in HST3168 and 40 bp in BSVK22. The assembled mitogenomes covered 27% of the mitochondrial genome for both species in the HST3168 library and 59% (*C. crocuta*) and 81% (*C. antiquitatis*) in the BSVK22 library. aDNA damage patterns were in accordance with the expected patterns of DNA degradation in terms of high proportions of C > T transitions at the 5′- and G > A transitions at the 3′-ends (electronic supplementary material, figure S1). *D. sumatrensis* is the closest extant relative of *C. antiquitatis* [16] and the extant spotted hyena is considered conspecific to the cave hyena [3]; compared with respective modern mitogenomes (NCBI accessions CM018432.1 and MF066642.1, respectively), the retrieved ancient mitogenomes showed shifts in nucleotide composition with higher proportions of G and T bases, and lower proportions of A and C (electronic supplementary material, figure S1). aDNA damage patterns showed the expected pattern regarding the *C. crocuta* mitogenomes, whereas this result was somewhat less consistent for the *C. antiquitatis* mitogenomes. This discrepancy is likely due to the markedly higher divergence of *C. antiquitatis* from the modern reference *D. sumatrensis*, compared to the cave hyena and its conspecific modern reference. Cytosine deamination due to aDNA degradation (i.e. high C > T substitution frequencies) indicates that DNA molecules are indeed ancient [17]. Considering such DNA decay-mediated substitutions, phylogenetic results must be interpreted with caution; however, integrating presumed aDNA decay-mediated substitutions in phylogenetic models may produce misleading results [18,19]. As the phylogenies produced here might be confounded by this, and the mitochondrial genome of the specimen from the Hohlenstein-Stadel cave is highly fragmented, we conducted further analyses only on the more complete mitogenome from the Bockstein site. Based on Bayesian inference conducted identically to a previous study [11], and including those sequences, the Bockstein sequence appeared to be substantially divergent from the previously published Siberian sequences, which were grouped into a common clade. The divergence time estimate of the European sequence was between >2 Mya and about 150 ka BP, while the two recovered Siberian clades split at a substantially younger time (figure 1). This high divergence and the inferred timing of the split suggest that the *C. antiquitatis* mitochondrial genome from Bockstein has been separated for a very long time from Siberian populations, which in contrast do not seem to display long-lasting phylogeographic patterns [12]. This contrasts with the hypothesis of repeated range expansions into Western Europe during cold stages of the Late Pleistocene, at least for the mitochondrial lineage of our sample.

As this result is only based on a single sample from a hyena coprolite, we refrain from any further interpretation. However, the mitogenome assemblies produced here are the first mitogenomic records of European woolly rhinoceros and are thus an important resource to help resolve the phylogeography of this iconic Pleistocene megafauna species. The fact that these were retrieved with relative ease from a coprolite of another species (i.e. no remain associated directly with woolly rhinoceros was needed) stresses the value of obtaining genomic data from a wide range of materials. As with these samples, many archaeological objects retrieved in past excavations and existing in collections, are to date a largely overlooked source of ancient DNA.

## Data Availability

Raw sequence data of the hybridization capture were made available as an NCBI BioProject (PRJNA933601) [20]. All details of raw data format and content are provided in the supplementary materials. Mitogenome assemblies were made available on Figshare: https://doi.org/10.6084/m9.figshare.22144169.v1 [21]. The data are provided in the electronic supplementary material, [22].
